# How international doctoral students’ fields of study, proficiency in English and gender interact with their sense of making progress in English academic writing abilities

**DOI:** 10.1371/journal.pone.0296186

**Published:** 2023-12-22

**Authors:** Wai Mar Phyo, Marianne Nikolov, Ágnes Hódi

**Affiliations:** 1 Doctoral School of Education, University of Szeged, Szeged, Hungary; 2 Department of English Applied Linguistics, University of Pécs, Pécs, Hungary; 3 Department of Kindergarten Teacher Training, University of Szeged, Szeged, Hungary; Golestan University, ISLAMIC REPUBLIC OF IRAN

## Abstract

This study investigates how non-native English-speaking (NNES) doctoral students self-assess their English academic writing (EAW) abilities. A total of 255 international NNES students, hailing from 49 different countries and speaking 48 mother tongues, voluntarily participated in our study. They were enrolled in 65 PhD programs at 14 universities across Hungary during the 2021–2022 academic year. To address our research aim, we developed a survey using a 6-point Likert scale, following the guidelines of Dörnyei and Dewaele (2022). The survey focused on self-assessing their abilities to write academic texts in English. The analysis results indicate that students lacked confidence in their EAW abilities at the beginning of their PhD studies but exhibited increased confidence at the current stage. The results also highlight the influence of gender and English language proficiency on EAW self-assessments. Additionally, senior PhD students demonstrated greater confidence in field-specific lexical knowledge compared to their first-year peers. This study highlights the fact that NNES novice writers lacked the necessary EAW skills upon entering their PhD programs, making it challenging for them to start doctoral-level writing immediately. This underscores the need for comprehensive support that encompasses both enhancing English language proficiency and providing academic writing assistance.

## 1. Introduction

Effective scholarly writing in English at the doctoral level presents a considerable challenge, especially for individuals with limited prior exposure to academic discourse [1–3]. This complexity is acknowledged by Hyland [4–6], who defined English for academic purposes as “an approach to language education based on identifying the specific language features, discourse practices, and communicative skills of target academic groups, and which recognizes the subject-matter needs and expertise of learners” [4] (pp. 383–384). The central endeavor of a doctoral student revolves around the timely submission of a dissertation. Hence, proficient English academic writing (EAW) skills are indispensable in doctoral education, acting as a cornerstone for success on the journey of obtaining a doctoral degree [7]. EAW skills are pivotal throughout academic studies, crucial for writing a doctoral thesis, engaging in scholarly discussions, disseminating research findings, and nurturing critical thinking. Mastery of EAW empowers doctoral candidates to communicate their research effectively, contribute to scholarly dialogues, and extend the impact of their findings. In a continuously evolving landscape of doctoral education with a global orientation, nurturing robust EAW abilities remains crucial, enabling aspirants to flourish as proficient scholars and researchers in their specialized domains [1, 8].

The process of doctoral writing, regarded as a distinctive literary genre, operates on the foundations of disciplinary epistemic practices [9]. This exacting endeavor has been noted as a universal concern, as indicated by [10] practices [11] referring to it as "something everyone is worried about." Beyond the creation of a thesis, doctoral candidates must fulfill publication prerequisites by their thesis defense, consequently magnifying the challenges of EAW for non-native English-speaking (NNES) novice writers [1, 12, 13].

The complexity of academic writing at the doctoral level is amplified by its distinct audience: subject experts within specialized research fields [14]. As a consequence, doctoral students are expected to present information with precision, aligning their work with discipline-specific norms. The responsibility to avoid ambiguity in their textual content underscores their obligation to adhere to rigorous standards [15]. Due to its demanding nature, doctoral writing has been studied by many researchers and it has been approached from different perspectives, including supervisory [16–18], pedagogical [2, 3, 19–24], and contextual perspectives ([25–30], as well as what challenges they pose [1, 23, 31], how they impact students’ well-being [32, 33] and development over time [34, 35]. However, very few studies have explored how NNES doctoral students’ self-perceived their EAW abilities at the starting point and at a later point in their PhD studies. The present study aims to fill this gap. It also aims to examine how students’ fields of study, proficiency in English and gender interact with their EAW abilities.

More specifically, no study has been conducted in a context like Hungary, where English is used as a lingua franca for both faculty and students. While Hungarian is the official language of the country, the prominence of English has grown due to its status as an international language of academia and global communication [36, 37]. Doctoral students studying in Hungary need to accomplish two phases in their PhD programs. The initial phase encompasses fulfilling oral and written course requisites and presenting oral and written progress reports on their literature review and empirical research each semester in their respective doctoral programs. Subsequently, in the second phase, they disseminate their research through academic engagements such as international conferences, publications, and completion of their dissertations. These pursuits require a proficient command of English academic writing skills. It is evident that writing academic papers at the expected level of their academic communities demands extra effort and time from NNES students. We believe that our findings offer invaluable insights into the EAW experiences of NNES doctoral students, thereby informing stakeholders, including the students themselves, tutors in doctoral programs, and developers of PhD curricula.

## 2. An overview of the theoretical framework

Scholarly writing at the doctoral level was described as a “grueling” experience [38]. “Along the way to a finished thesis, students can become mired in uncertainty about what they are discovering–intellectually stuck–and then lose confidence in their ability to express themselves within an academically accepted writing style” [39] (p.140). Insufficient knowledge about academic writing conventions tends to be an issue for uncompletion of doctoral dissertations [40]. Researchers have explored challenges NNES graduate students may face in academic writing; here we overview the most relevant ones.

Studies conducted by Lin and Morrison [23], Ma [41] and Wang et al. [23, 42, 43] identified vocabulary as one of the challenging factors in discipline-oriented scholarly writing. Ability to use appropriate vocabulary efficiently is a concern in EAW, as the quality of academic texts can be low due to lexical limitations such as “lack of vocabulary, repetition of words, incorrect usage of words, avoiding complex and complicated words” [44] (p.252). Therefore, vocabulary choice and its appropriateness in context are often a challenge for students at the postgraduate level [23, 42, 43, 45–47]. A good command of grammar is another skill students should be equipped with, as poor grammar competency leads to confusion, distraction and ambiguity and it may seriously harm the validity of statements writers want to make in their scholarly texts [31, 48, 49]. Ramírez-Castañeda [50] stated that doctoral students’ poor grammatical competency was one of the reasons given by reviewers why they rejected manuscripts.

Citation, a vital skill in academic writing, is another area doctoral students often struggle with. How to refer to others’ work is challenging for novice writers and studies have proven that doctoral students need to learn both the convention and function of citing [51, 52]. In all types of academic texts, all sources from which information, theories and ideas are taken, have to be clearly cited in order to meet ethical standards of scholarship and to avoid plagiarism. As Lin and Morrison [23] pointed out, postgraduate students need to improve their knowledge in terms of writing in-text citations and entering references in an academic manner according to the requirements they are expected to meet following the preferred referencing style of journals and respective doctoral institutions.

Paraphrasing is another aspect of area students may find difficult [23, 53–55]. Although paraphrasing is an effective writing strategy used to synthesize significant information from multiple sources, it requires the writer to be well-equipped with a thorough understanding of what they have read beyond linguistic interpretation [56]. This issue is also addressed in a study conducted by Ma [42]: “being able to understand concepts in readings and integrate those concepts in one’s own writing” is a challenge” (p.1185). Therefore, how well students can paraphrase largely depends on both their linguistic knowledge and a deep level of understanding of their respective fields. As authors are responsible for not distorting the original meaning, the ability to paraphrase precisely is a key area and it may pose a challenge for novice writers.

Moreover, it is necessary to apply a critical approach and tone when summarizing key points and drawing conclusions. Doctoral students are expected to be able to analyze complex ideas critically to ensure that their contributions fill important gaps in their disciplinary fields. However, criticality is an essential skill many novices may lack [1], as “critical engagement with textual forms also requires cultural engagement with broader knowledge-creating and knowledge-producing forms” [57] (p.4). According to Bruce [58], students should develop critical competence to “innovate, challenge, resist or reshape the discourses of their own academic community”. In the study conducted by Kotamjani et al. [59], the “ability to write critically and be inquisitive and critical of their writing process” was mentioned as “one of the most problematic academic writing areas” (p.193), which point is in line with previous studies [1, 50, 55, 60].

A doctoral dissertation itself is a genre of scholarly writing; its scope is expected to deal with a specific focus [9]. Therefore, the ability to present ideas in a logical fashion in a dissertation while transparently describing all the steps in the research along the rationales underlying them is another area students often find challenging. For example, Ankawi [53] highlighted that Arabic postgraduate students often struggled when they tried to “clarify their points” (p. 121). This issue emerged in multiple other studies [31, 54, 59]; for example, Kotamjani et al. [59] also remarked that focused instruction on this area was necessary for students to make progress with their scholarly writing. The ability to present ideas in a logical sequence, in a manner the readers can follow without difficulty, can also be negatively affected by limitations in lexis, grammar and discourse competence [31, 42, 60, 61].

Literature reviews are parts of scholarly texts, including doctoral dissertations. Overviews of previous work are to cover all the academic sources directly related to one’s own research [62–65]. In some cases, students fail to demonstrate the link between their work and previous literature clearly; instead, they convolute ideas together [66]. Doctoral students tend to spend hundreds of hours working on a literature review section; however, when they are not well-informed about the approach to writing up a critical review, they may end up with “frustration and delay” [67] (p.1). Therefore, writing a critical literature review is also a skill doctoral students should develop.

Findings of the critical literature review are expected to support the problems students examine in their research. Problem statements in doctoral dissertations must demonstrate a clear relationship with previous research and be supported by evidence [65, 67, 68]. Therefore, in developing clear problem statements, doctoral students need to have the ability to use precise key terms, provide citations appropriately, and know how to present ideas logically in order to highlight a missing gap that needs to be fulfilled [63, 67, 69].

For PhD students, it is important to know how to search for closely related literature prior to their writing. Almatarneh et al. [44] reported that novice scholars often struggled with writing a literature review due to their limited understanding of how to find relevant literature. Similarly, Walter and Stouck [70] observed that students had difficulty retrieving necessary academic sources. Moreover, NNES students struggle with information overload, as the vast amount of available literature can make it challenging to identify the most relevant sources [67]. Limited time and resources intensify this difficulty, impeding their ability to navigate databases and critically evaluate sources [38, 71, 72].

In addition, doctoral students need to effectively respond to and interact with their supervisors’ comments during the writing process. This interaction forms a crucial facet of the supervisory relationship, facilitating the growth and refinement of the student’s research and writing abilities [18, 73–75]. Constructive feedback from supervisors serves as a compass guiding the trajectory of the student’s work. It is through these interactions that students gain valuable insights, refine their arguments, and enhance the overall quality of their scholarly texts. Moreover, the dynamic exchange of ideas and revisions fosters a deeper understanding of academic standards and expectations, ultimately contributing to the production of high-quality research dissemination [74, 76, 77].

In conclusion, navigating scholarly writing at the doctoral level is a formidable journey characterized by a range of challenges. Novice writers often grapple with a lack of knowledge in academic conventions. The demanding nature of doctoral writing, underscored by vocabulary, grammar, citation, paraphrasing, criticality, and logical presentation challenges, demands a comprehensive set of EAW skills. Ensuring precision in diction, grammar, and discourse competence is not only critical for logical presentation but also for crafting a clear problem statement supported by evidence. The intricate interplay of these challenges underscores the multifaceted nature of EAW competence and its pivotal role in producing valuable contributions to the scholarly community.

## 3. Method

### 3.1. Research design

The current study is part of a larger project using an exploratory sequential mixed methods design, as proposed by Creswell and Creswell [62]. This research design involves a two-phase process: an initial qualitative phase followed by a quantitative phase. The current study focused on the quantitative phase. The exploratory sequential mixed methods design has been recognized for its strength in providing a holistic understanding of complex research topics [62].

### 3.2. Research questions

RQ1. How do doctoral students from non-native English-speaking backgrounds perceive their English academic writing abilities at the start of their PhD studies?
RQ2. How do they self-assess their EAW abilities at the current point in their PhD studies?
RQ3. What is the difference between their self-assessments at the start and now?
RQ4. Are there significant differences between male and female participants’ self-assessments?
RQ5. What is the difference between different proficiency levels in students’ self-assessments?
RQ6. How do students’ self-assessments compare in their 1^st^, 2^nd^, 3^rd^, 4^th^ and 4+ years of PhD studies?
RQ7. To what extent do students’ self-assessments differ across the fields of their PhD programs?

### 3.3. Participants

A total of 255 NNES volunteers participated in the survey. The survey was conducted using a convenience sampling method, where participants were recruited based on their accessibility and willingness to participate [78]. While this method offers several strengths, such as cost-effectiveness and ease of recruitment, it is important to acknowledge potential limitations, including the risk of selection bias, as individuals who volunteered may differ in certain characteristics from those who did not.

The survey included inquiries related to the participants’ demographic particulars, including gender, doctoral programs, universities, countries of origin, mother tongues, current academic semester, English proficiency level upon entering the PhD program, and the name of the English exam undertaken. Analysis of the dataset revealed a gender distribution of 125 females (49%), 127 males (49.80%), and 3 (1.17%) unspecified. Participants enrolled in 65 doctoral programs conducted in English across 14 universities in Hungary during the 2021–2022 academic year. Coming from 49 diverse countries, they represented 48 first languages. Notably, participants’ English proficiency levels varied: 22 students (8.6%) passed C2 proficiency level exams, the highest standard according to the *Common European Framework of Reference for Languages* (*CEFR*) [79]. Additionally, 118 students (46.3%) claimed to have C1 proficiency (also advanced level), whereas 115 (45.1%) claimed to be at B2 (upper-intermediate) proficiency level. Their distribution across various years of their PhD studies was as follows: 1st-year (36.5%), 2nd-year (25.1%), 3rd-year (18%), 4th-year (16.9%), 4+ years (2%), 1.6% not indicate their status. Top of Form Participants’ research areas were as follows: (1) agricultural science (10.6%), (2) computer science and information technology (5.1%), (3) economic science (8.6%), (4) educational science (24.3%), (5) engineering science (14.9%), (6) medical and health science (7.5%), (7) natural science (9.4%) and (8) humanities (19.6%).

### 3.4. Data collection instrument

To investigate how NNES doctoral students assessed their English academic writing abilities, based on the findings of the literature review, a survey was developed, focusing on two constructs: EAW abilities at the start of the PhD program (EAWS: 17 items) and EAW ability at the current point/now (EAWN: 22 items). For comparisons, we included 14 identical statements in both thematic units (i.e., EAWS and EAWN). Students were asked to assess their abilities on a 6-point Likert Scale (1 = strongly disagree, 2 = disagree, 3 = slightly disagree, 4 = slightly agree, 5 = agree, 6 = strongly agree) in order to avoid neutral answers following Dörnyei and Dewaele [80].

#### 3.4.1. Reliability and validity

To assess the reliability of the instrument, Cronbach’s Alpha (α) was used. The guidelines proposed by [81, 82] were followed to evaluate the internal consistency. According to these guidelines, an α value of ≥ 0.9 is considered excellent, a value ranging from 0.9 to 0.8 is good, from 0.8 to 0.7 is acceptable, from 0.7 to 0.6 is questionable, from 0.6 to 0.5 is poor, and a value below 0.5 is deemed unacceptable. The first construct, EAWS of 17 items, exhibited a high reliability coefficient (α = 0.942). The other construct, EAWN comprising 22 items, showcased excellent reliability (α = 0.979).

To assess the instrument’s construct validity, a confirmatory factor analysis (CFA) was conducted, following the guidelines of [83]. CFA is recommended when researchers possess a thorough understanding of the scale, encompassing variables or factors, item correlations, and factor interrelationships. The Kaiser-Meyer-Olkin (KMO) test resulted in a value of 0.933, indicating high suitability for factor analysis. The test of sphericity yielded a p-value of <0.001, further supporting the adequacy of factor analysis. In CFA, the fitness indices have specific cutoff values for acceptability: χ2 should be insignificant; TLI, and CFI should be ≥ 0.90; and SRMR and RMSEA should be ≤ 0.10 [84]. In this study, the goodness of fit was achieved with X2/df < 5, RMSEA < 0.08, CFI and TLI > 0.90, SRMR < 0.08, and nearly the smallest AIC and BIC values.

To assess the convergent validity of the constructs, the study employed the Average Variance Extracted (AVE) and Composite Reliability (CR) measures. According to [85–87], AVE values should exceed 0.5 for each composite construct, whereas the acceptable range for CR is typically between 0.70 and 0.80, with values above 0.80 considered good, and values above 0.90 considered excellent. The results showed strong convergent validity, as evidenced by the high AVE values and CR values (see Table 1).

**Table 1:** Reliability and validity indicators of the constructs.

Constructs	Alpha	CR	AVE
English academic writing at start of PhD studies (EAWS)	0.942	0.950	0.532
English academic writing now (EAWN)	0.979	0.981	0.701

### 3.5. Ethical approval certificate from the Institutional Review Board (IRB)

Prior to data collection, the research proposal underwent a rigorous ethical review process, seeking approval from the Institutional Review Board (IRB) of the Doctoral School of Educational Sciences at the University of Szeged. The application included an outline of the research plan, explicitly delineating key aspects such as voluntary participation, implementation of anonymized coding, utilization of inclusive language in presenting research outcomes, and the commitment to using the data solely for research purposes. Following a comprehensive evaluation by the IRB, the research project was granted ethical approval (ref #: 17/2021; see S1 Appendix). As a next step, a survey link was generated using Google Forms. Outreach to NNES PhD students enrolled in Hungarian institutions was conducted through multiple channels. The survey link was posted on platforms such as PhD students’ forums on Facebook and Messenger groups, Stipendium-Hungaricum newsletter, PhD students’ Google group and WhatsApp group. Furthermore, we sent representatives of the PhD student body an email with the survey link. The survey message explicitly stated that participation was entirely voluntary, and participants were assured that their data would be coded and used exclusively for the purpose of informing stakeholders about the challenges and requirements faced by doctoral students in EAW (see the survey in S2 Appendix). The researchers’ contact information, including affiliation and email address, were available to facilitate participant communication at any time. The survey was accessible from 2/21/2022 to 12/10/2022. Half of the participants provided their email address volunteering to participate in follow-up interviews.

### 3.6. Data analysis

First, to ensure the privacy of the participants, students’ responses were coded, and all personally identifiable information was anonymized following [88]. Using Statistical Packages for Social Sciences (SPSS) and Excel software, statistical analyses were conducted to gain insights from the dataset, aligning with established methodologies [84, 86]. The analysis of participants’ perceptions concerning each statement was done by descriptive analyses. Furthermore, to discern potential disparities in responses across genders, independent samples t-tests were done. To gauge how respondents’ perceived abilities changed from the commencement to the present stage of the PhD journey, paired samples t-tests were used. The application of one-way ANOVA enabled a comprehensive comparison of student cohorts based on their proficiency levels, academic years, and age groups, as recommended in the literature [76, 84, 86].

## 4. Results of the data analysis

### 4.1. Students’ self-assessed abilities at the start of their doctoral studies

As shown in Table 2, the highest mean was found on the item about knowing *how to write a literature review in English* (M = 4.48, SD = 1.32). Students also believed that they knew *how to write a research paper in English* (M = 4.23, SD = 1.56) when they started their doctoral studies. In line with those self-assessments, the responses also reflect that they *had experience in English academic writing* (M = 4.44, SD = 1.51). They agreed that they were familiar with *guidelines like APA or MLA* (M = 4.12, SD = 1.58) and *citing and referencing sources* (M = 4.14, SD = 1.58), however, standard deviations were high, indicating important differences in the population. These results show that students tended to be confident about their English academic writing abilities at the beginning of their PhD studies.

**Table 2:** Students’ self-assessment at the start of their PhD studies.

	English academic writing abilities at the start (EAWS)	M	SD	Differences based on gender				Differences based on proficiency			
					M	SD	*p*		M	SD	*p*
EAWS1	My special English vocabulary was not good enough to write my course assignments.	4.16	1.47	Female	4.08	1.53	0.378	C2	5.05	1.40	0.000**
				Male	4.24	1.42		C1	4.66	1.39	
								B2	3.51	1.30	
EAWS2	I knew how to write a literature review in English.	4.48	1.32	Female	4.23	1.41	0.005**	C2	5.00	1.51	0.004**
				Male	4.70	1.18		C1	4.66	1.29	
								B2	4.20	1.25	
EAWS3	I did not know how to write a research paper in English.	4.23	1.56	Female	3.96	1.66	0.008**	C2	4.23	1.74	0.356
				Male	4.48	1.42		C1	4.38	1.60	
								B2	4.09	1.48	
EAWS4	I was familiar with guidelines like APA or MLA.	4.12	1.58	Female	3.88	1.63	0.015*	C2	4.73	1.64	0.007**
				Male	4.36	1.48		C1	4.32	1.51	
								B2	3.80	1.57	
EAWS5	I had no experience in English academic writing.	4.44	1.51	Female	4.40	1.59	0.589	C2	4.77	1.34	0.000**
				Male	4.50	1.46		C1	4.88	1.44	
								B2	3.96	1.49	
EAWS6	I could write so that my audience understood the meaning clearly.	4.75	1.09	Female	4.66	1.11	0.22	C2	5.32	1.13	0.000**
				Male	4.83	1.09		C1	4.92	1.09	
								B2	4.48	1.03	
At the beginning of the program, when I wrote in English, I had no difficulties with											
EAWS7	paraphrasing texts	3.78	1.55	Female	3.64	1.60	0.119	C2	4.50	1.60	0.000**
				Male	3.94	1.50		C1	4.30	1.48	
								B2	3.14	1.36	
EAWS8	citing and referencing sources	4.14	1.58	Female	3.93	1.68	0.033*	C2	4.73	1.42	0.002**
				Male	4.35	1.47		C1	4.39	1.58	
								B2	3.78	1.54	
EAWS9	organizing paragraphs	3.96	1.50	Female	3.83	1.55	0.132	C2	4.50	1.63	0.000**
				Male	4.12	1.46		C1	4.38	1.46	
								B2	3.45	1.37	
EAWS10	grammar	4.06	1.48	Female	4.00	1.57	0.445	C2	5.23	1.11	0.000**
				Male	4.14	1.37		C1	4.49	1.42	
								B2	3.41	1.30	
EAWS11	special vocabulary	3.60	1.43	Female	3.54	1.52	0.409	C2	4.64	1.43	0.000**
				Male	3.69	1.34		C1	4.03	1.38	
								B2	2.98	1.21	
EAWS12	writing paragraphs	4.16	1.44	Female	4.07	1.49	0.262	C2	5.00	1.35	0.000**
				Male	4.28	1.38		C1	4.58	1.34	
								B2	3.60	1.34	
EAWS13	presenting ideas logically	3.93	1.42	Female	3.82	1.50	0.214	C2	4.36	1.71	0.000**
				Male	4.05	1.34		C1	4.32	1.32	
								B2	3.47	1.34	
EAWS14	stating problems clearly	3.80	1.43	Female	3.60	1.48	0.038	C2	4.23	1.74	0.000**
				Male	3.98	1.38		C1	4.19	1.36	
								B2	3.32	1.31	
EAWS15	summarizing key points	3.97	1.47	Female	3.76	1.53	0.029*	C2	4.50	1.63	0.000**
				Male	4.17	1.41		C1	4.30	1.39	
								B2	3.54	1.41	
EAWS16	drawing conclusions	3.98	1.39	Female	3.68	1.42	0.001**	B3	4.77	1.45	0.000**
				Male	4.27	1.31		B4	4.22	1.32	
								B5	3.60	1.34	
EAWS17	being critical	3.55	1.53	Female	3.30	1.58	0.008**	B6	4.27	1.75	0.000**
				Male	3.80	1.45		B7	3.83	1.54	
								B8	3.15	1.37	

* Statistically significant at the p < .05 level.

** Statistically significant at the p < .01 level.

Students were also confident about their linguistic and discourse competences: especially about *grammar* (M = 4.06, SD = 1.48) and *writing paragraphs* (M = 4.16, SD = 1.44). The highest mean was found on the item claiming that they *could write* so *that their audience understood the meaning clearly* (M = 4.75, SD = 1.09). They also believed that their *vocabulary was good enough for writing course assignments* (M = 4.16, SD = 1.47). On the rest of the items in Table 2, students’ self-assessed mean scores ranged from 3.55 to 3.98 (SD between 1.39 to 1.55), indicating that they agreed with the given statements to a lesser degree on the 6-point Likert Scale. The lowest self-assessed mean score was found for the item *being critical* (M = 3.55, SD = 1.53).

### 4.2 Students’ self-assessed abilities at the current point of their doctoral studies

As Table 3 shows, the mean scores for the self-assessed items at the current point of studies tended to be higher, whereas the SD data were lower (means ranged between 4.25 and 5.02, standard deviations ranged between 0.94 and 1.14). Therefore, the results revealed that students agreed with all the statements to a high extent (see Table 2). The item on *citing and referencing sources* received the highest mean score (M = 5.02, SD = 0.96) and the self-assessed score for *using guidelines like APA or MLA* was also high (M = 4.82, SD = 1.20), indicating that students’ self-assessments were consistent. Respondents agreed with all the statements to a large degree, except for the item *Errors are rare in my texts* which received the lowest mean score (M = 4.25, SD = 1.21), indicating that NNES students are still less confident about the accuracy of their English texts than in other aspects of EAW.

**Table 3:** Students’ self-assessment at the current point of their PhD studies.

	English academic writing abilities now (EAWN)	M	SD	Differences based on gender				Differences based on proficiency			
					M	SD	*p*		M	SD	*p*
EAWN1	I can write clear, highly accurate and smoothly complex academic texts.	4.49	1.14	Female	4.31	1.22	0.011*	C2	5.09	1.15	0.000**
				Male	4.68	1.03		C1	4.67	1.08	
								B2	4.21	1.12	
EAWN2	I can show flexibility in formulating ideas in differing linguistic forms to convey meaning precisely.	4.57	1.09	Female	4.39	1.16	0.007**	C2	5.00	1.11	0.000**
				Male	4.76	1.00		C1	4.82	0.99	
								B2	4.25	1.09	
EAWN3	I have a good command of specific vocabulary related to my larger field of study.	4.82	0.97	Female	4.74	1.06	0.168	C2	5.45	0.80	0.000**
				Male	4.91	0.88		C1	4.97	0.85	
								B2	4.55	1.03	
EAWN4	I can create coherent and cohesive texts.	4.70	0.99	Female	4.52	1.07	0.004**	C2	5.32	0.72	0.000**
				Male	4.88	0.88		C1	4.84	0.95	
								B2	4.45	1.00	
EAWN5	I can use a wide range of connectors and other cohesive devices.	4.72	1.02	Female	4.70	1.11	0.687	C2	5.50	0.60	0.000**
				Male	4.75	0.93		C1	4.93	0.95	
								B2	4.37	1.01	
EAWN6	I can demonstrate consistent and highly accurate grammatical control of complex language forms.	4.56	1.05	Female	4.42	1.14	0.031*	C2	5.32	0.95	0.000**
				Male	4.70	0.95		C1	4.85	0.95	
								B2	4.12	0.98	
EAWN7	Errors are rare in my texts.	4.25	1.21	Female	4.18	1.23	0.362	C2	5.05	0.95	0.000**
				Male	4.32	1.18		C1	4.53	1.06	
								B2	3.83	1.25	
EAWN8	I can write clear, smoothly flowing, complex texts.	4.62	0.99	Female	4.49	1.07	0.037*	C2	5.23	0.69	0.000**
				Male	4.75	0.89		C1	4.78	0.94	
								B2	4.34	1.00	
EAWN9	I can write a critical overview of the relevant literature.	4.64	0.99	Female	4.50	1.06	0.013*	C2	5.32	0.78	0.000**
				Male	4.80	0.89		C1	4.80	0.91	
								B2	4.35	1.01	
EAWN10	I can write a publishable paper on an empirical study I designed and implemented.	4.65	1.00	Female	4.43	1.09	0.000**	C2	5.23	0.92	0.000**
				Male	4.88	0.86		C1	4.80	0.88	
								B2	4.40	1.07	
Now, when I write in English, I have no difficulties with											
EAWN11	paraphrasing texts	4.58	1.13	Female	4.46	1.21	0.124	C2	5.18	1.01	0.000**
				Male	4.69	1.06		C1	4.88	0.97	
								B2	4.16	1.17	
EAWN12	citing and referencing sources	5.02	0.96	Female	4.87	1.13	0.013*	C2	5.64	0.58	0.000**
				Male	5.17	0.75		C1	5.12	0.93	
								B2	4.80	0.98	
EAWN13	organizing paragraphs	4.85	1.00	Female	4.70	1.11	0.022*	C2	5.41	1.01	0.000**
				Male	4.99	0.86		C1	5.03	0.92	
								B2	4.56	0.99	
EAWN14	grammar	4.66	1.09	Female	4.53	1.17	0.030*	C2	5.32	0.95	0.000**
				Male	4.82	0.94		C1	4.95	0.93	
								B2	4.25	1.11	
EAWN15	special vocabulary	4.65	1.08	Female	4.56	1.15	0.168	C2	5.18	1.10	0.000**
				Male	4.75	1.00		C1	4.91	0.94	
								B2	4.30	1.09	
EAWN16	writing paragraphs	4.79	1.06	Female	4.64	1.16	0.022*	C2	5.23	1.19	0.000**
				Male	4.94	0.94		C1	5.02	0.91	
								B2	4.48	1.09	
EAWN17	presenting ideas logically	4.78	0.96	Female	4.56	1.06	0.000**	C2	5.23	0.75	0.000**
				Male	5.00	0.81		C1	4.97	0.83	
								B2	4.50	1.04	
EAWN18	stating problems clearly	4.78	0.94	Female	4.62	1.02	0.005**	C2	5.41	0.67	0.000**
				Male	4.94	0.83		C1	4.94	0.83	
								B2	4.50	1.00	
EAWN19	summarizing key points	4.86	0.96	Female	4.68	1.03	0.004**	C2	5.32	0.72	0.000**
				Male	5.03	0.86		C1	5.05	0.82	
								B2	4.58	1.04	
EAWN20	drawing conclusions	4.80	1.00	Female	4.58	1.05	0.001**	C2	5.23	0.87	0.000**
				Male	5.01	0.92		C1	5.00	0.84	
								B2	4.52	1.10	
EAWN21	being critical	4.68	1.02	Female	4.45	1.13	0.000**	C2	5.18	0.80	0.000**
				Male	4.91	0.86		C1	4.87	0.89	
								B2	4.39	1.11	
EAWN22	using guidelines like APA or MLA	4.82	1.20	Female	4.79	1.26	0.587	C2	5.41	1.01	0.005**
				Male	4.87	1.13		C1	4.93	1.26	
								B2	4.60	1.12	

* Statistically significant at the p < .05 level.

** Statistically significant at the p < .01 level.

### 4.3 Differences between self-assessed scores at the start and now

The survey comprised 14 identical items at the start (EAWS2, EAWS3, EAWS4, EAWS7 to EAWS17) and at the current point (EAWN9, EAWN10, EAWN11 to EAWN22) for the purpose of comparison. According to the results presented in Tables 2 and 3, the self-assessed scores are higher at the current point (Table 3), compared to the scores at the start (Table 2). Therefore, we conducted paired samples *t*-tests for those identical self-assessments in order to answer research question 3. The results confirmed that the self-assessed scores at the current point were significantly higher at the level of p = 0.000, except the pair about *writing a literature review* which shows its statistical significance at the p < .05 level (see Table 4).

**Table 4:** Differences between self-assessed scores at the start and now.

Pairs	M	SD	SE	95% Confidence Interval of the Difference		t (254)	p
				Lower	Upper		
EAWS2-EAWN9 (writing a literature review)	-0.16078	1.24295	0.07784	-0.31407	-0.00750	-2.066	0.040
EAWS3-EAWN10(writing research findings)	-0.501961	1.584558	0.099229	-0.697377	-0.306545	-5.059	0.000
EAWS4-EAWN22(using guidelines such as APA, MLA)	-0.70196	1.40772	0.08816	-0.87557	-0.52835	-7.963	0.000
EAWS7-EAWN11(paraphrasing texts)	-0.79216	1.38323	0.08662	-0.96274	-0.62157	-9.145	0.000
EAWS8-EAWN12(citing and referencing sources)	-0.88235	1.34643	0.08432	-1.04840	-0.71630	-10.465	0.000
EAWS9-EAWN13(organizing paragraphs)	-0.88235	1.27432	0.07980	-1.03951	-0.72520	-11.057	0.000
EAWS10-EAWN14(grammar)	-0.60000	1.15561	0.07237	-0.74252	-0.45748	-8.291	0.000
EAWS11-EAWN15(vocabulary)	-1.04706	1.29115	0.08085	-1.20629	-0.88783	-12.950	0.000
EAWS12-EAWN16(writing paragraphs)	-0.62353	1.25782	0.07877	-0.77865	-0.46841	-7.916	0.000
EAWS13-EAWN17 (presenting ideas logically)	-0.84314	1.18675	0.07432	-0.98949	-0.69678	-11.345	0.000
EAWS 14-EAWN18(stating the problems clearly)	-0.98431	1.24220	0.07779	-1.13751	-0.83112	-12.654	0.000
EAWS15-EAWN19(summarizing key points)	-0.89020	1.29646	0.08119	-1.05008	-0.73031	-10.965	0.000
EAWS16-EAWN20(drawing conclusions)	-0.81569	1.16433	0.07291	-0.95928	-0.67210	-11.187	0.000
EAWS17-EAWN21(being critical)	-1.12549	1.29807	0.08129	-1.28557	-0.96541	-13.846	0.000

### 4.4. Gender differences at the start and now

Descriptive analysis results showed that male students’ self-assessed scores were higher on all statements both at the start and at the current point. We conducted independent samples *t*-tests to investigate if the results were significant. As shown in Table 1, the gender difference was statistically significant for 7 out of 17 self-assessed items at the start of the students’ PhD studies (EAWS 2–4, 8, 15–17). However, the differences between female and male students were significant for 16 out of 22 items at the present point (EAWN 1, 2, 6, 8–21) on the respondents’ PhD journey in Table 3.

### 4.5. Differences across proficiency levels at entry to doctoral programs

The descriptive analysis results showed that the lowest scores on all self-assessed statements concerned only the B2 group, whereas the C2 level group received the highest self-assessed scores, and the C1 group, the second highest. To investigate if the differences are statistically significant among the three groups (C2, C1, B2), we performed One-way ANOVA tests. The results at the start are shown in Table 2 and at the current point in Table 3. The test revealed that differences were significant both at the start and at the current moment, except for one statement: *I did not know how to write a research paper in English* (at the start). For this statement, the difference is not significant at the p < .05 level for any of the three proficiency levels [F (2, 252) = 1.038, p = .365]. According to Post Hoc Tukey HSD tests, the scores of C2 and C1 groups were statistically higher than those at B2 level (p < .05) both at the start and now. Moreover, the significant difference at the p < .05 level was found between C2 and C1 groups for two self-assessed items: *I can use a wide range of connectors and other cohesive devices* and *citing and referencing sources* (see details in Tables 1 and 2 in the S3 Appendix).

### 4.6 Differences across the years

To investigate whether there are significant differences across five different years (1^st^, 2^nd^, 3^rd^, 4^th^, 4+ year of PhD studies), we performed one-way ANOVA tests for all the self-assessed items in the English academic writing abilities scale at the current moment (22 self-assessed items). The analysis showed a significant difference for only one item: *I have a good command of specific vocabulary related to my larger field of study* at the p<0.05 level among the different groups of PhD studies [F (4, 246) = 3.036, p = 0.018]. Therefore, we conducted a Post Hoc comparison using a Tukey HSD test. We found that 3^rd^-year PhD (M = 5.07, SD = 0.93) and 4^th^-year PhD (M = 5.09, SD = 1.00) students’ self-assessed scores were significantly higher than those of 1^st^-year PhD (M = 4.59, SD = 1.02) students, which makes perfect sense. Students’ scope of academic vocabulary directly related to their specialized field is developing over the years as they manage to fulfill their EAW tasks (see details for the students’ self-assessed scores across the years in Table 3 in the S3 Appendix).

### 4.7. Differences across the research fields of the PhD programs

In order to examine the difference across the fields, first, we performed one-way ANOVA tests for the self-assessed scores at the start. We found a statistically significant difference for one item *(I was familiar with guidelines like APA or MLA)* across the fields [F (7, 247) = 3.066, p = 0.004]. According to a Post Hoc comparison using Tukey HSD test, the mean score of Humanities (M = 4.38, SD = 1.51), Educational science (M = 4.20, SD = 1.47), Agricultural Science (M = 4.85, SD = 1.29) were significantly higher than that of Computer Science and Information Technology (M = 2.69, SD = 1.65). According to the descriptive statistics at the current point, we found that the group of Computer Science and Information Technology students gave the lowest self-assessed scores for 18 out of 22 items (see Table 4 in S3 Appendix). However, when we conducted one-way ANOVA tests to investigate the differences across the scientific fields of the participants’ doctoral programs, we did not find significant differences (p>.05).

## 5. Discussion

Understanding NNES doctoral students’ EAW experience has significant implications for both academic institutions and the students themselves. This discussion section sheds light on the pivotal insights gained from the study on how 225 NNES doctoral students assessed their EAW abilities at two stages of their academic journey. The importance of comprehending these self-assessments lies in the fact that EAW skills are fundamental for scholarly communication and success in the international academic arena. EAW abilities impact not only successful completion of PhDs, but they also contribute to careers in the global academic community.

The study was guided by seven research questions inquiring into key aspects of NNES doctoral students’ self-assessed EAW abilities. From the outset, a clear pattern emerged from the descriptive analysis (RQ1), revealing consistency in self-assessed scores for writing *a literature review* and *a research paper* and having *experience in English academic writing*. Students’ highest self-assessed mean score concerned writing *a literature review* at the start of PhD studies; therefore, we inferred that they must have had experience in writing this genre before they applied to a PhD program. Although participants came from very different educational backgrounds, one thing in common for them was that they all had at least a master’s degree, as it is a prerequisite to PhD entry in Hungary. As a literature review is a required chapter in a thesis, we conclude that their highest self-assessed scores for writing a *literature review* were based on their previous experience. This point explains the high mean scores on the two statements about citations at the start of their PhD studies (*I was familiar with guidelines like APA or MLA; citing and referencing sources*). Another key finding related to the students’ self-assessments at the start was that lower self-assessed scores were found on the items which required linguistic and discourse competence, and a high level of critical reasoning (*paraphrasing texts*; *organizing paragraphs*; *presenting ideas logically*; *summarizing key points*; *drawing conclusions*; *being critical*), whereas higher self-assessed scores were found on the items that do not rely on critical thinking, but mostly linguistic and discourse competence (*vocabulary for writing course assignments*; *grammar*; *writing paragraphs*; *citations and references*; *ability to write so that the audience understood the meaning clearly*). These findings indicate that participants were aware that writing well requires not only writing skills, but also the ability to reason critically. Previous studies also found that the ability to paraphrase, summarize and draw conclusions focusing on what is most relevant to one’s own research requires a high level of criticality; similarly, presenting ideas and paragraphs in a logical sequence also requires critical thinking [1, 47, 55, 58, 59, 65, 89]. In line with these results, we found the lowest mean score on *being critical*, indicating that students were the least satisfied with their critical ability at the start. This result impacted their self-assessment on other items which require criticality.

In terms of the students’ self-assessments at the current point of PhD studies (RQ2), the mean scores confirmed that they were confident about their EAW abilities. The highest score was found on the self-assessed item which does not need a deep level of critical thinking skill (*citing and referencing sources*), revealing that students know how to use citations to support their statements with research-based evidence.

The analysis of the third research question (RQ3) revealed a notable trend among NNES doctoral students: a sense of progress in their writing abilities. This outcome underscores the students’ capacity to advance their writing skills at their individual pace. Furthermore, this result underscores they were not only honing their writing skills but also bolstering their self-assurance in the process. Another inference drawn from the findings pertaining to RQ3 is that the students perceived a positive trajectory in their EAW development over the course of their PhD studies. This evolution was accompanied by an increase in their self-confidence levels, further underlining the transformative impact of their doctoral education on their EAW skills and self-perceived capabilities.

Regarding gender differences (RQ 4), male students reported significantly higher self-assessed scores compared to their female peers across all the statements. This outcome aligns with prior research, which has consistently highlighted a divergence between male and female students in terms of academic achievement and their own perceived capabilities. Typically, females tend to exhibit lower confidence in their abilities when compared to their male peers [90–97].

An additional finding underscores that higher proficiency in the target language (RQ 5) correlates positively with an increased sense of confidence in the students’ capacity to compose academic texts in English. Notably, English language proficiency emerged as a pivotal factor that consistently underpins doctoral students’ academic achievement in numerous prior studies, particularly in instances where English serves as the medium in international scholarly communication [98–103].

Addressing the variability in students’ self-perceptions across the five academic year categories (RQ 6), our analysis found statistically significant differences among students in their 1^st^, 2^nd^, 3^rd^, 4^th^, 4+ years concerning a specific item. Notably, the quality of academic writing is profoundly contingent upon proficiency in the target language, as reported in the previous literature [23, 41, 52, 104]. Given that students’ language proficiency levels varied upon entering their PhD programs, it follows that this divergence played a significant role in shaping their perceptions of EAW abilities. This observation helps explain the phenomenon where participants in more advanced PhD programs (3rd-, 4th-, and 4+ year PhD students) displayed no significantly greater confidence in their EAW abilities compared to their junior peers.

While no statistically significant differences were found across specific fields of study (RQ 7), a pronounced trend emerged on students enrolled in Computer Science and Information Technology programs. They appeared to lack adequate exposure to EAW upon starting their PhD programs. Specifically, this subgroup had limited familiarity with the prevalent referencing styles, such as *APA or MLA*. This pattern persisted at the current stage of assessment, where the lowest self-assessed scores were consistently attributed to this group. This observation aligns with earlier research that revealed a similar trend among PhD students in Computer Studies. They were often found unprepared to meet requirements in doctoral-level writing upon entering PhD programs, primarily due to their undergraduate degrees not requiring writing academic texts [105–107].

In conclusion, these findings illuminate the landscape of NNES doctoral students’ EAW abilities and their interactions with diverse factors. By grasping the complex nature of EAW proficiency, academic institutions can foster an inclusive environment that empowers novice writers to excel in EAW and socialize into the global academic discourse.

## 6. Conclusion

In this study, we investigated the self-assessed EAW abilities of international doctoral students of NNES backgrounds, who were pursuing academic programs in Hungary. We examined the interactions across factors such as gender, proficiency levels, years of study, and academic disciplines on their self-assessment scores.

A consistent pattern emerged throughout the study regarding NNES doctoral students’ initial self-assessments of their EAW skills. Initially, students expressed lower confidence in their EAW abilities at the outset of their PhD studies. However, as their doctoral journey progressed, there was a noticeable enhancement in their self-perceived EAW abilities, underscoring the efficacy of doctoral education in developing EAW proficiency.

While factors such as English proficiency background and gender impacted students’ self-perceived EAW abilities, the shared trajectory of growth among NNES doctoral students was particularly noteworthy. These findings highlight the important role of doctoral education in fostering self-confidence among academic writers.

Our conclusions align with a metaphorical analysis study conducted in the same context [108–110]., where students vividly portrayed the rewarding nature of their academic writing process. Despite acknowledging the demanding nature of the endeavor, our participants echoed a sense of accomplishment and advancement in their EAW abilities. Moreover, in the previous studies involving the same students, they demonstrated proactive management of their progress in both EAW abilities and research knowledge throughout their academic years in the PhD program [109, 110]. To sum up, our study underscores the transformative impact of doctoral education on NNES doctoral students’ EAW abilities.

## 7. Implications

The current study’s findings underscore critical implications for the support and development of NNES doctoral students in their EAW) abilities. It reveals a common deficiency among NNES doctoral students upon entering the PhD program, emphasizing the necessity for targeted support to elevate their EAW skills to the required scholarly level.

Given that doctoral programs often lack explicit training in academic writing, relying on research students’ independent work, curriculum developers face a pivotal consideration. Integration of specialized EAW courses within doctoral programs becomes imperative. These courses would extend beyond educational assistance, encompassing essential writing support, including access to editing and proofreading services. Notably, such courses must be thoughtfully designed by respective doctoral institutions, acknowledging the discipline-specific nature of EAW at the doctoral level. In doing so, each discipline should engage an experienced scholar within their field to oversee the editing of their PhD students’ writing.

Moreover, the implications extend beyond institutional considerations. Novice NNES scholars aspiring to join English-medium international doctoral programs receive a clear message: active cultivation of their EAW abilities is paramount. This proactive approach prepares them to effectively navigate the challenges inherent in academic writing at the doctoral level, ensuring a smoother and more successful academic journey.

In conclusion, our findings call for a comprehensive and discipline-specific approach to supporting NNES doctoral students in their EAW abilities, urging both educational institutions and aspiring scholars to actively contribute to and enhance the scholarly landscape.

## 8 Limitations and future directions

While this study offers valuable insights into the EAW abilities of NNES doctoral students, there are several limitations that warrant consideration. First, data collection relied on a voluntary participation approach, leading to a sample size of 255 NNES international PhD students. This might introduce potential selection bias, as students already burdened with their doctoral responsibilities might not have volunteered. Consequently, those who perceive themselves as struggling or contemplating dropping out of their program might be underrepresented. Furthermore, it is essential to acknowledge that all participants in this study were enrolled within the PhD education system of Hungary. In addition, the findings might not be universally applicable to NNES doctoral students in varying educational contexts.

To enhance the applicability of future research, it is important to broaden the participant pool to include a more diverse range of NNES doctoral students from various educational backgrounds and regions. By doing so, a more comprehensive understanding of the challenges and progress in EAW abilities could be gained. Additionally, building upon the indication that the students in this study are making progress, it would be beneficial to investigate the factors contributing to this progress more deeply. Furthermore, in light of the promising progress exhibited by the students in this study, it is essential to conduct a more in-depth investigation into the factors underpinning this advancement. This exploration can extend to include the perspectives of educators, teachers, and supervisors. Additionally, delving into instructors’ proficiency in assessing academic writing can provide valuable insights. This research direction holds the potential to provide actionable insights for both students and educators, facilitating the cultivation of stronger EAW abilities among NNES doctoral candidates.

## Supporting information

S1 Appendix (PDF)

S2 Appendix Survey of non-native speaker doctoral students’ English academic writing experiences. (PDF)

S3 Appendix (PDF)
