# Supplementary material for: How international doctoral students’ fields of study, proficiency in English and gender interact with their sense of making progress in English academic writing abilities
Source: PLoS One. 2023 Dec 22;18(12):e0296186. doi: 10.1371/journal.pone.0296186 (PMC10745157; doi:10.1371/journal.pone.0296186)
Supplement: S1 Appendix — (PDF) [file pone.0296186.s001.pdf]

University of Szeged

Institutional Review Board
Doctoral School of Education

6722 Szeged, 30-34 Petőfi S. Av., Hungary
Phone/fax: +36 62 544-032

Wai Mar Phyo
PhD Student: Doctoral School of Education
Reference number: 17/2021
Subject: Ethical evaluation of a research project

Date: 2 November, 2021

ETHICAL APPROVAL

The Insitutional Review Board (IRB) of the Doctoral School of Education, University of Szeged has recently reviewed your application for an ethical approval (Title of the Research Project: "**How PhD students cope with academic writing requirements: A study of PhD students in the Doctoral School of Educational Sciences at the University of Szeged**", supervisors: Dr. Marianne Nikolov and Dr. Ágnes Hódi). This proposal is deemed to meet the requirements of the ethical conducts on social research with human subjects of the Doctoral School of Education, University of Szeged.

IRB decision: approved

Justification:
The research project meets the requirements of the professional-ethical criteria of the social research including human subjects within the field of education science. Main goal of the study is to examine the English academic writing challenges that PhD students at the Doctoral School of Educational Sciences, University of Szeged, and analyze their influencing factors. Participants are 50 PhD students as well as their supervisors and tutors. No students under the age of 18 will participate in the study. Data are collected via Interviews and Questionnaires and Analyses of students' academic written texts by using the tool for the automatic analysis of lexical sophistication (TAALES) before and after receiving feedback from supervisors and reviewers. Participation is voluntary and anonymous. Informed consent from the participants will be asked. Procedure of the data collection does not harm their privacy law, it does not have an impact on the participants' mental or physical health. Data cannot be handled by persons to whom they are not concerned.

In a summary, full ethical approval has been granted.

We wish you all the best for the conduct of the project.

Prof. Dr. Bettina Pikó
IRB coordinator
