# Supplementary material for: How international doctoral students’ fields of study, proficiency in English and gender interact with their sense of making progress in English academic writing abilities
Source: PLoS One. 2023 Dec 22;18(12):e0296186. doi: 10.1371/journal.pone.0296186 (PMC10745157; doi:10.1371/journal.pone.0296186)
Supplement: S2 Appendix — (PDF) [file pone.0296186.s002.pdf]

Appendix B

Survey of non-native speaker doctoral students' English Academic Writing experiences

Dear Doctoral Student,

I'm conducting a survey of doctoral students' experiences whose programs are conducted in English and their first language is ***not*** English. I'm a member of this group.

Please fill in this questionnaire. I'd like to learn about your journey in academic English writing, from the beginning of your studies to where you are now. This survey is anonymous. Data will be coded and used for research purposes only to inform stakeholders about the needs and challenges doctoral students face when using academic English. It will take a few minutes to fill in this survey. If you want to learn about the findings, please add your email address (this is optional). You can contact me by email any time.
Thank you for your cooperation.

Author
Author's department
Author's university
Author's email address

1. Name of your doctoral program and school
2. Name of your university
3. Which semester are you in now?
4. What is your first language?
5. What is your country of origin?
6. Gender
7. Email address (optional)

**When I started the doctoral program**,						
	1 Strongly Disagree	2 Disagree	3 Slightly disagree	4 Slightly agree	5 Agree	6 Strongly Agree
1. My special English vocabulary was not good enough to write my course assignments.						
2. I knew how to write a literature review in English.						
3. I did not know how to write a research paper in English.						
4. I was familiar with guidelines like APA or MLA.						
5. I had no experience in English academic writing.						
6. I could write so that my audience understood the meaning clearly.						
**At the beginning of the program, when I wrote in English, I had difficulties with**						
7. paraphrasing texts						
8. citing and referencing sources						
9. organizing paragraphs						

10. grammar						
11. special vocabulary						
12. writing paragraphs						
13. presenting ideas logically						
14. stating problems clearly						
15. summarizing key points						
16. drawing conclusions						
17. being critical						

**At this point in my doctoral studies**						
	1 Strongly Disagree	2 Disagree	3 Slightly disagree	4 Slightly agree	5 Agree	6 Strongly Agree
18. I can write clear, highly accurate and smoothly flowing complex academic texts.						
19. I can show flexibility in formulating ideas in differing linguistic forms to convey meaning precisely.						
20. I have a good command of specific vocabulary related to my larger field of study.						
21. I can create coherent and cohesive texts.						
22. I can use a wide range of connectors and other cohesive devices.						
23. I can demonstrate consistent and highly accurate grammatical control of complex language forms.						
24. Errors are rare in my texts.						
25. I can write clear, smoothly flowing, complex texts.						
26. I can write a critical overview of the relevant literature.						
27. I can write a publishable paper on an empirical study I designed and implemented.						
**Now, when I write in English, I have no difficulties with**						
28. paraphrasing texts						
29. citing and referencing sources						
30. organizing paragraphs						
31. grammar						
32. special vocabulary						
33. writing paragraphs						
34. presenting ideas logically						
35. stating problems clearly						
36. summarizing key points						
37. drawing conclusions						
38. being critical						
39. using guidelines like APA or MLA						
