# Supplementary material for: How international doctoral students’ fields of study, proficiency in English and gender interact with their sense of making progress in English academic writing abilities
Source: PLoS One. 2023 Dec 22;18(12):e0296186. doi: 10.1371/journal.pone.0296186 (PMC10745157; doi:10.1371/journal.pone.0296186)
Supplement: S3 Appendix — (PDF) [file pone.0296186.s003.pdf]

Appendix C

Table 1. Differences at the start of PhD studies based on proficiency levels

Tukey HSD

			Mean Difference (I-J)	Std. Error	Sig.	95% Confidence Interval	
						Lower Bound	Upper Bound
My special English vocabulary was not good enough to write my course assignments.	B2	C2	-1.533*	0.314	0.000	-2.27	-0.79
		C1	-1.151*	0.177	0.000	-1.57	-0.73
I knew how to write a literature review in English.	B2	C2	-.80342*	0.30085	0.022	-1.5127	-0.0941
		C1	-.46721*	0.16963	0.017	-0.8671	-0.0673
I did not know how to write a research paper in English.	B2	C2	-.80342*	0.30085	0.022	-1.5127	-0.0941
		C1	-.46721*	0.16963	0.017	-0.8671	-0.0673
I was familiar with guidelines like APA or MLA.	B2	C2	-.92385*	0.36037	0.029	-1.7735	-0.0742
		C1	-.51555*	0.20319	0.031	-0.9946	-0.0365
I had no experience in English academic writing.	B2	C2	-.81546*	0.33795	0.044	-1.6122	-0.0187
		C1	-.92205*	0.19055	0.000	-1.3713	-0.4728
I could write so that my audience understood the meaning clearly.	B2	C2	-.83955*	0.24768	0.002	-1.4235	-0.2556
		C1	-.44378*	0.13965	0.005	-0.773	-0.1145
paraphrasing texts	B2	C2	-1.36325*	0.33309	0.000	-2.1486	-0.5779
		C1	-1.16497*	0.18781	0.000	-1.6078	-0.7222
citing and referencing sources	B2	C2	-.94949*	0.35981	0.024	-1.7978	-0.1012
		C1	-.61015*	0.20287	0.008	-1.0885	-0.1319
organizing paragraphs	B2	C2	-1.04701*	0.33311	0.005	-1.8324	-0.2617
		C1	-.92632*	0.18782	0.000	-1.3691	-0.4835
grammar	C2	C1	.73589*	0.31184	0.050	0.0007	1.4711
	B2	C2	-1.81702*	0.31162	0.000	-2.5517	-1.0823
		C1	-1.08112*	0.1757	0.000	-1.4954	-0.6669
special vocabulary	B2	C2	-1.65346*	0.30458	0.000	-2.3716	-0.9354
		C1	-1.05158*	0.17174	0.000	-1.4565	-0.6467
writing paragraphs	B2	C2	-1.40171*	0.31137	0.000	-2.1358	-0.6676
		C1	-.97930*	0.17556	0.000	-1.3932	-0.5654
presenting ideas logically	B2	C2	-.89355*	0.31649	0.014	-1.6397	-0.1474
		C1	-.84888*	0.17845	0.000	-1.2696	-0.4282
stating problems clearly	B2	C2	-.90249*	0.31849	0.014	-1.6534	-0.1516
		C1	-.86487*	0.17958	0.000	-1.2882	-0.4415
summarizing key points	B2	C2	-.96154*	0.33027	0.011	-1.7402	-0.1829
		C1	-.76326*	0.18622	0.000	-1.2023	-0.3242
drawing conclusions	B2	C2	-1.17444*	0.31128	0.001	-1.9083	-0.4406
		C1	-.62585*	0.17551	0.001	-1.0396	-0.2121
being critical	B2	C2	-1.12743*	0.345	0.004	-1.9408	-0.314
		C1	-.68229*	0.19453	0.002	-1.1409	-0.2237

*. The mean difference is significant at the 0.05 level.

Table 2. Differences at the current point in PhD studies based on proficiency levels

Tukey HSD

			Mean Difference (I-J)	Std. Error	Sig.	95% Confidence Interval	
						Lower Bound	Upper Bound
I can write clear, highly accurate and smoothly flowing complex academic texts.	B2	C2	-.88578*	0.25626	0.002	-1.49	-0.2816
		C1	-.46729*	0.14449	0.004	-0.8079	-0.1266
I can show flexibility in formulating ideas in differing linguistic forms to convey meaning precisely.	B2	C2	-.75214*	0.24364	0.006	-1.3266	-0.1777
		C1	-.57110*	0.13737	0.000	-0.895	-0.2472
I have a good command of specific vocabulary related to my larger field of study.	B2	C2	-.90754*	0.21682	0.000	-1.4187	-0.3964
		C1	-.42713*	0.12225	0.002	-0.7153	-0.1389
I can create coherent and cohesive texts.	B2	C2	-.86519*	0.2228	0.000	-1.3905	-0.3399
		C1	-.38322*	0.12562	0.007	-0.6794	-0.087
I can use a wide range of connectors and other cohesive devices.	C2	C1	.56897*	0.22223	0.030	0.045	1.0929
		B2	1.13248*	0.22208	0.000	0.6089	1.6561
	B2	C2	-1.13248*	0.22208	0.000	-1.6561	-0.6089
		C1	-.56351*	0.12522	0.000	-0.8587	-0.2683
I can demonstrate consistent and highly accurate grammatical control of complex language forms.	B2	C2	-1.19852*	0.22374	0.000	-1.726	-0.671
		C1	-.73379*	0.12615	0.000	-1.0312	-0.4364
Errors are rare in my texts.	B2	C2	-1.21639*	0.2653	0.000	-1.8419	-0.5909
		C1	-.69680*	0.14959	0.000	-1.0495	-0.3441
I can write clear, smoothly flowing, complex texts.	B2	C2	-.88539*	0.22121	0.000	-1.4069	-0.3638
		C1	-.43398*	0.12473	0.002	-0.728	-0.1399
I can write a critical overview of the relevant literature.	B2	C2	-.96775*	0.21999	0.000	-1.4864	-0.4491
		C1	-.45130*	0.12404	0.001	-0.7437	-0.1589
I can write a publishable paper on an empirical study I designed and implemented.	B2	C2	-.82556*	0.22604	0.001	-1.3585	-0.2927
		C1	-.40001*	0.12745	0.005	-0.7005	-0.0995
paraphrasing texts	B2	C2	-1.01943*	0.24821	0.000	-1.6046	-0.4342
		C1	-.71692*	0.13995	0.000	-1.0469	-0.387
citing and referencing sources	C2	C1	.51567*	0.21722	0.048	0.0036	1.0278
	B2	C2	-.83294*	0.21707	0.000	-1.3447	-0.3212
		C1	-.31727*	0.12239	0.027	-0.6058	-0.0287
organizing paragraphs	B2	C2	-.84499*	0.22345	0.001	-1.3718	-0.3182
		C1	-.46176*	0.12599	0.001	-0.7588	-0.1647
grammar	B2	C2	-1.07032*	0.23617	0.000	-1.6271	-0.5135
		C1	-.70041*	0.13316	0.000	-1.0144	-0.3865
special vocabulary	B2	C2	-.88267*	0.23864	0.001	-1.4453	-0.32
		C1	-.60603*	0.13456	0.000	-0.9233	-0.2888
writing paragraphs	B2	C2	-.74864*	0.23741	0.005	-1.3084	-0.1889
		C1	-.53861*	0.13386	0.000	-0.8542	-0.223
presenting ideas logically	B2	C2	-.72300*	0.21563	0.003	-1.2314	-0.2146
		C1	-.46124*	0.12158	0.001	-0.7479	-0.1746
stating problems clearly	B2	C2	-.90482*	0.20864	0.000	-1.3967	-0.4129

		C1	-.43538*	0.11764	0.001	-0.7127	-0.158
summarizing key points	B2	C2	-.73699*	0.21462	0.002	-1.243	-0.231
		C1	-.47053*	0.12101	0.000	-0.7558	-0.1852
drawing conclusions	B2	C2	-.70591*	0.22539	0.005	-1.2373	-0.1745
		C1	-.47863*	0.12708	0.001	-0.7782	-0.179
being critical	B2	C2	-.78866*	0.22971	0.002	-1.3302	-0.2471
		C1	-.47753*	0.12952	0.001	-0.7829	-0.1722
using guidelines like APA or MLA	B2	C2	-.81080*	0.27343	0.009	-1.4554	-0.1662
		C1	-0.33274	0.15417	0.081	-0.6962	0.0307

*. The mean difference is significant at the 0.05 level.

Table 3. Students' self-assessments across the years

		Mean	Std. Deviation
I can write clear, highly accurate and smoothly flowing complex academic texts.	First-year PhD	4.32	1.24
	Second-year PhD	4.42	0.99
	Third-year PhD	4.70	1.11
	Fourth-year PhD	4.77	1.13
	8+ semester	4.20	0.84
I can show flexibility in formulating ideas in differing linguistic forms to convey meaning precisely.	First-year PhD	4.48	1.19
	Second-year PhD	4.55	0.89
	Third-year PhD	4.76	1.08
	Fourth-year PhD	4.60	1.16
	8+ semester	4.60	1.14
I have a good command of specific vocabulary related to my larger field of study.	First-year PhD	4.59	1.02
	Second-year PhD	4.80	0.84
	Third-year PhD	5.07	0.93
	Fourth-year PhD	5.09	1.00
	8+ semester	5.00	0.71
I can create coherent and cohesive texts.	First-year PhD	4.57	0.98
	Second-year PhD	4.70	0.87
	Third-year PhD	4.85	1.13
	Fourth-year PhD	4.86	1.06
	8+ semester	4.40	0.55
I can use a wide range of connectors and other cohesive devices.	First-year PhD	4.57	1.03
	Second-year PhD	4.78	0.97
	Third-year PhD	4.78	1.11
	Fourth-year PhD	4.95	1.00
	8+ semester	4.40	0.55
I can demonstrate consistent and highly accurate grammatical control of complex language forms.	First-year PhD	4.41	1.11
	Second-year PhD	4.56	0.92
	Third-year PhD	4.63	1.14

	Fourth-year PhD	4.79	1.04
	8+ semester	4.60	0.55
Errors are rare in my texts.	First-year PhD	4.15	1.27
	Second-year PhD	4.25	1.10
	Third-year PhD	4.35	1.29
	Fourth-year PhD	4.44	1.18
	8+ semester	4.00	1.22
I can write clear, smoothly flowing, complex texts.	First-year PhD	4.54	0.89
	Second-year PhD	4.47	0.98
	Third-year PhD	4.72	1.20
	Fourth-year PhD	4.93	0.94
	8+ semester	4.60	1.14
. I can write a critical overview of the relevant literature.	First-year PhD	4.53	0.95
	Second-year PhD	4.64	1.07
	Third-year PhD	4.80	0.88
	Fourth-year PhD	4.77	1.04
	8+ semester	4.60	1.14
I can write a publishable paper on an empirical study I designed and implemented.	First-year PhD	4.46	0.96
	Second-year PhD	4.63	1.00
	Third-year PhD	4.89	0.88
	Fourth-year PhD	4.88	1.20
	8+ semester	4.80	0.84
paraphrasing texts	First-year PhD	4.45	1.15
	Second-year PhD	4.53	1.10
	Third-year PhD	4.70	1.26
	Fourth-year PhD	4.86	1.01
	8+ semester	4.60	0.89
citing and referencing sources	First-year PhD	4.92	0.96
	Second-year PhD	4.95	0.97
	Third-year PhD	5.26	0.88
	Fourth-year PhD	5.16	1.00
	8+ semester	4.60	1.14
organizing paragraphs	First-year PhD	4.68	0.97
	Second-year PhD	4.77	1.00
	Third-year PhD	5.09	1.07
	Fourth-year PhD	5.12	0.93
	8+ semester	4.80	0.84
grammar	First-year PhD	4.62	1.08
	Second-year PhD	4.63	0.97
	Third-year PhD	4.65	1.30
	Fourth-year PhD	4.84	1.04
	8+ semester	4.60	1.14

special vocabulary	First-year PhD	4.49	1.04
	Second-year PhD	4.64	1.01
	Third-year PhD	4.80	1.19
	Fourth-year PhD	4.93	1.06
	8+ semester	4.80	0.84
writing paragraphs	First-year PhD	4.67	1.04
	Second-year PhD	4.72	1.11
	Third-year PhD	4.98	1.02
	Fourth-year PhD	5.00	1.07
	8+ semester	4.80	0.84
presenting ideas logically	First-year PhD	4.68	0.87
	Second-year PhD	4.77	1.05
	Third-year PhD	4.87	1.00
	Fourth-year PhD	4.98	0.96
	8+ semester	4.80	0.84
stating problems clearly	First-year PhD	4.67	0.84
	Second-year PhD	4.78	1.00
	Third-year PhD	4.85	1.01
	Fourth-year PhD	5.00	0.98
	8+ semester	4.80	0.84
summarizing key points	First-year PhD	4.72	0.90
	Second-year PhD	4.83	1.05
	Third-year PhD	5.07	0.88
	Fourth-year PhD	5.05	1.00
	8+ semester	4.80	0.84
drawing conclusions	First-year PhD	4.70	0.95
	Second-year PhD	4.72	1.08
	Third-year PhD	5.00	0.99
	Fourth-year PhD	5.00	1.02
	8+ semester	4.60	0.55
being critical	First-year PhD	4.54	1.01
	Second-year PhD	4.72	1.11
	Third-year PhD	4.83	0.88
	Fourth-year PhD	4.84	1.11
	8+ semester	4.40	0.55
using guidelines like APA or MLA	First-year PhD	4.65	1.22
	Second-year PhD	4.83	1.24
	Third-year PhD	4.98	1.09
	Fourth-year PhD	5.02	1.22
	8+ semester	4.60	1.14

Table 4. Students' self-assessed scores across the fields

		N	Mean	Std. Deviation
I can write clear, highly accurate and smoothly flowing complex academic texts.	Agricultural Science	27	5.04	0.90
	Computer Science and Information Technology	13	3.92	1.19
	Economic Science	22	4.41	1.33
	Educational science	62	4.37	1.01
	Engineering Science	38	4.47	0.95
	Medical and Health Science	19	4.53	1.43
	Natural Science	24	4.46	1.25
	Humanities	50	4.56	1.20
I can show flexibility in formulating ideas in differing linguistic forms to convey meaning precisely.	Agricultural Science	27	5.04	0.81
	Computer Science and Information Technology	13	3.92	1.26
	Economic Science	22	4.50	1.14
	Educational science	62	4.48	0.95
	Engineering Science	38	4.55	1.01
	Medical and Health Science	19	4.84	1.21
	Natural Science	24	4.54	1.41
	Humanities	50	4.56	1.09
I have a good command of specific vocabulary related to my larger field of study.	Agricultural Science	27	5.19	0.68
	Computer Science and Information Technology	13	4.31	0.95
	Economic Science	22	4.41	1.37
	Educational science	62	4.81	0.92
	Engineering Science	38	4.79	0.84
	Medical and Health Science	19	5.00	1.20
	Natural Science	24	4.79	0.88
	Humanities	50	4.92	0.94
I can create coherent and cohesive texts.	Agricultural Science	27	5.07	0.73
	Computer Science and Information Technology	13	4.15	1.07
	Economic Science	22	4.59	1.05
	Educational science	62	4.66	0.90
	Engineering Science	38	4.71	0.90
	Medical and Health Science	19	4.53	1.50
	Natural Science	24	4.67	0.96
	Humanities	50	4.82	0.98
I can use a wide range of connectors and other cohesive devices.	Agricultural Science	27	5.04	0.76
	Computer Science and Information Technology	13	4.31	0.95
	Economic Science	22	4.36	1.22
	Educational science	62	4.74	0.97
	Engineering Science	38	4.79	0.99
	Medical and Health Science	19	4.63	1.30
	Natural Science	24	4.63	0.97
	Humanities	50	4.82	1.02
	Agricultural Science	27	4.89	0.85

	Computer Science and Information Technology	13	4.15	1.07
I can demonstrate consistent and highly accurate grammatical control of complex language forms.	Economic Science	22	4.18	1.37
	Educational science	62	4.58	0.98
	Engineering Science	38	4.50	0.89
	Medical and Health Science	19	4.58	1.26
	Natural Science	24	4.50	1.18
	Humanities	50	4.68	1.00
Errors are rare in my texts.	Agricultural Science	27	4.37	1.33
	Computer Science and Information Technology	13	4.15	1.14
	Economic Science	22	4.14	1.17
	Educational science	62	4.16	1.10
	Engineering Science	38	4.39	1.03
	Medical and Health Science	19	4.00	1.56
	Natural Science	24	4.25	1.36
	Humanities	50	4.36	1.26
I can write clear, smoothly flowing, complex texts.	Agricultural Science	27	4.96	0.71
	Computer Science and Information Technology	13	4.38	0.65
	Economic Science	22	4.41	1.22
	Educational science	62	4.52	0.90
	Engineering Science	38	4.74	0.89
	Medical and Health Science	19	4.47	1.26
	Natural Science	24	4.71	1.04
	Humanities	50	4.62	1.10
I can write a critical overview of the relevant literature.	Agricultural Science	27	4.85	0.91
	Computer Science and Information Technology	13	4.00	1.00
	Economic Science	22	4.64	1.09
	Educational science	62	4.44	1.00
	Engineering Science	38	4.84	0.82
	Medical and Health Science	19	4.79	1.18
	Natural Science	24	4.63	1.06
	Humanities	50	4.74	0.92
I can write a publishable paper on an empirical study I designed and implemented.	Agricultural Science	27	4.93	0.92
	Computer Science and Information Technology	13	4.31	0.95
	Economic Science	22	4.59	1.10
	Educational science	62	4.53	0.97
	Engineering Science	38	4.84	0.82
	Medical and Health Science	19	4.74	1.52
	Natural Science	24	4.67	0.92
	Humanities	50	4.60	0.99
paraphrasing texts	Agricultural Science	27	4.85	1.13
	Computer Science and Information Technology	13	4.23	1.01
	Economic Science	22	4.45	1.26
	Educational science	62	4.40	1.05
	Engineering Science	38	4.74	0.92

	Medical and Health Science	19	4.58	1.54
	Natural Science	24	4.58	1.10
	Humanities	50	4.66	1.21
citing and referencing sources	Agricultural Science	27	5.37	0.63
	Computer Science and Information Technology	13	4.85	0.80
	Economic Science	22	4.95	1.00
	Educational science	62	4.85	0.90
	Engineering Science	38	5.13	0.84
	Medical and Health Science	19	5.00	1.33
	Natural Science	24	5.25	0.74
	Humanities	50	4.92	1.18
organizing paragraphs	Agricultural Science	27	5.07	0.92
	Computer Science and Information Technology	13	4.69	0.75
	Economic Science	22	4.73	1.24
	Educational science	62	4.79	0.89
	Engineering Science	38	4.95	0.84
	Medical and Health Science	19	4.89	1.10
	Natural Science	24	4.79	1.10
	Humanities	50	4.82	1.16
grammar	Agricultural Science	27	4.96	0.94
	Computer Science and Information Technology	13	4.31	1.18
	Economic Science	22	4.45	1.22
	Educational science	62	4.66	0.90
	Engineering Science	38	4.58	1.15
	Medical and Health Science	19	4.58	1.39
	Natural Science	24	4.75	1.19
	Humanities	50	4.72	1.07
	Total	255	4.66	1.09
special vocabulary	Agricultural Science	27	4.81	1.04
	Computer Science and Information Technology	13	4.38	0.87
	Economic Science	22	4.23	1.34
	Educational science	62	4.53	1.02
	Engineering Science	38	4.87	0.96
	Medical and Health Science	19	4.79	1.27
	Natural Science	24	4.75	1.11
	Humanities	50	4.70	1.05
writing paragraphs	Agricultural Science	27	5.04	0.85
	Computer Science and Information Technology	13	4.62	0.87
	Economic Science	22	4.77	1.19
	Educational science	62	4.68	1.00
	Engineering Science	38	4.89	0.92
	Medical and Health Science	19	4.79	1.44
	Natural Science	24	4.67	1.20
	Humanities	50	4.82	1.10
presenting ideas logically	Agricultural Science	27	5.07	0.78

	Computer Science and Information Technology	13	4.46	0.88
	Economic Science	22	4.64	1.18
	Educational science	62	4.58	0.90
	Engineering Science	38	4.92	0.85
	Medical and Health Science	19	4.84	1.30
	Natural Science	24	4.83	0.92
	Humanities	50	4.84	0.98
stating problems clearly	Agricultural Science	27	5.00	0.96
	Computer Science and Information Technology	13	4.23	0.73
	Economic Science	22	4.55	1.01
	Educational science	62	4.65	0.85
	Engineering Science	38	4.92	0.88
	Medical and Health Science	19	4.95	1.22
	Natural Science	24	4.83	0.96
	Humanities	50	4.88	0.92
summarizing key points	Agricultural Science	27	5.11	0.85
	Computer Science and Information Technology	13	4.23	1.09
	Economic Science	22	4.82	0.91
	Educational science	62	4.76	0.92
	Engineering Science	38	4.95	0.80
	Medical and Health Science	19	5.00	1.20
	Natural Science	24	4.92	1.06
	Humanities	50	4.88	0.98
drawing conclusions	Agricultural Science	27	5.07	0.92
	Computer Science and Information Technology	13	4.38	1.04
	Economic Science	22	4.68	1.09
	Educational science	62	4.71	0.95
	Engineering Science	38	4.87	0.88
	Medical and Health Science	19	4.79	1.44
	Natural Science	24	4.92	0.97
	Humanities	50	4.82	0.98
being critical	Agricultural Science	27	5.07	0.87
	Computer Science and Information Technology	13	4.23	1.17
	Economic Science	22	4.91	1.06
	Educational science	62	4.44	0.90
	Engineering Science	38	4.68	0.84
	Medical and Health Science	19	4.79	1.32
	Natural Science	24	4.88	0.85
	Humanities	50	4.64	1.19
using guidelines like APA or MLA	Agricultural Science	27	5.04	1.16
	Computer Science and Information Technology	13	4.15	1.52
	Economic Science	22	5.00	1.15
	Educational science	62	4.94	0.88
	Engineering Science	38	4.71	1.16

Medical and Health Science	19	4.47	1.68
Natural Science	24	4.75	1.19
Humanities	50	4.90	1.28
